# Association between lower fasting plasma glucose levels during oral glucose tolerance test and adverse perinatal outcomes: A Chinese cohort study

**DOI:** 10.1371/journal.pmed.1004722

**Published:** 2025-09-23

**Authors:** Lulu Wang, Chao Tang, Mengqiu Cheng, Yanhui Hao, Siyue Chen, Siwei Zhang, Chen Zhang, Ben W. Mol, Yanting Wu, Hefeng Huang

**Affiliations:** 1 Institute of Reproduction and Development, Shanghai Key Laboratory of Reproduction and Development, Obstetrics and Gynecology Hospital, Fudan University, Shanghai, China; 2 Shanghai Key Lab of Female Reproductive Endocrine Related Diseases, Shanghai, China; 3 Department of Obstetrics and Gynecology, Monash University, Clayton, Australia; 4 Research Units of Embryo Original Diseases, Chinese Academy of Medical Sciences, Shanghai, China; 5 Key Laboratory of Reproductive Genetics (Ministry of Education), Department of Reproductive Endocrinology, Women’s Hospital, Zhejiang University School of Medicine, Hangzhou, China; Makerere University College of Health Sciences, UGANDA

## Abstract

**Background:**

It is unknown whether fasting plasma glucose (FPG) level within the normal range as defined by the International Association of Diabetes and Pregnancy Study Groups (IADPSG) criteria is associated with perinatal outcomes. This study explored the associations between FPG levels lower than the IADPSG threshold during oral glucose tolerance test (OGTT) and adverse perinatal outcomes in women with or without gestational diabetes mellitus (GDM).

**Methods and findings:**

From January 1, 2017, to May 31, 2022, this single-center retrospective cohort study included 33,417 women with singleton pregnancies at the Obstetrics and Gynecology Hospital of Fudan University, Shanghai, China. All women underwent a 75-g OGTT at 24–28 gestational weeks. The primary endpoint was a composite of adverse outcomes, including gestational hypertension, preeclampsia, fetal death and stillbirth, preterm birth, primary cesarean delivery, and small or large for gestational age. Overall, 3,108 (9.5%) women had IADPSG-defined GDM and of whom 2,426 (76.3%) had FPG levels below the IADPSG threshold. Compared to the GDM population, non-GDM women with borderline-normal FPG levels were at significantly greater risk of adverse outcomes with an adjusted odds ratio (aOR) of 1.62 (95% CI [1.20, 2.19]; *p* = 0.002) at 4.6 mmol/L, an aOR of 1.50 (95% CI [1.05, 2.13]; *p* = 0.025) at 4.8 mmol/L, and an aOR of 1.58 (95% CI [1.05, 2.40]; *p* = 0.030) at 4.9 mmol/L glucose level. Nonetheless, non-GDM women demonstrated significantly lower risk (aOR 0.66, 95% CI [0.44, 0.98]; *p* = 0.038) compared to GDM counterparts exhibiting low fasting glycemia at 3.9 mmol/L. However, this study was limited by its retrospective design and may lack generalizability to other ethnic groups.

**Conclusions:**

Even at FPG levels lower than the IADPSG threshold, FPG was significantly associated with adverse perinatal outcomes, and the associations presented different patterns in women with and without GDM.

## Author summary

**Why was this study done?:**

- Fasting plasma glucose (FPG) levels have been demonstrated to be associated with perinatal outcomes.
- Few studies have explored the associations between FPG levels below the International Association of the Diabetes and Pregnancy Study Groups (IADPSG) threshold and adverse perinatal outcomes.

**What did the researchers do and find?:**

- This single-center retrospective cohort study investigated the association between FPG levels lower than the IADPSG diagnostic threshold and adverse perinatal outcomes.
- Women without IADPSG-defined GDM who had borderline-normal FPG levels presented a greater risk of adverse outcomes.
- Women with IADPSG-defined GDM who had marked glucose fluctuations were found to be at an elevated risk of adverse outcomes.

**What do these findings mean?:**

- Even below the IADPSG diagnostic threshold, FPG levels were significantly associated with perinatal outcomes, with different patterns in women with and without GDM.
- Obstetric care should be tailored individually to women with different patterns of glucose metabolism.
- The main limitations of this study included its retrospective design and potential lack of generalizability to diverse ethnic populations.

## Abstract 3

In a retrospective analysis of fasting plasma glucose (FPG) levels in pregnant women in China, Lulu Wang and colleagues report that women with elevated FPG that is within the normal range are nevertheless at increased risk of adverse perinatal outcomes, indicating the need to individualize obstetric care rather than rely on diagnostic thresholds alone.

## Introduction

Gestational diabetes mellitus (GDM), defined as maternal hyperglycemia first recognized during pregnancy, affects approximately 14% of pregnant women globally [1,2]. GDM contributes to short-term perinatal complications, such as large for gestational age (LGA) and preterm delivery [3]. In the long term, women with GDM and their offspring are at increased risk of metabolic disorders and cardiovascular diseases [4–6].

Following the Hyperglycemia and Adverse Pregnancy Outcomes (HAPO) study, the diagnostic criteria for GDM proposed by the International Association of the Diabetes and Pregnancy Study Groups (IADPSG) gained worldwide endorsement, also in Asian populations [7–9]. A diagnosis of GDM is considered if any of the following thresholds are met: a fasting plasma glucose (FPG) ≥ 5.1 mmol/L, a 1-h plasma glucose ≥ 10.0 mmol/L, or a 2-h plasma glucose ≥ 8.5 mmol/L. China has adopted the IADPSG recommendations to perform a 75-g oral glucose tolerance test (OGTT) between 24 and 28 weeks of gestation [8].

However, FPG and post-load plasma glucose reflect distinct glucose metabolic statuses, leading to various perinatal complications [10,11]. Compared with post-load plasma glucose, FPG has been demonstrated to be a stronger predictor of perinatal outcomes and an independent risk factor for LGA [12–16]. Accumulating evidence suggests risks of short-term perinatal outcomes increase progressively with each grade of FPG elevation, including excessive birth weight, cesarean section, and abnormal biochemical traits in infants [7,17]. Recent large-scale cohort studies have also emphasized the association between isolated FPG levels during an OGTT and an increased long-term risk of type 2 diabetes [18,19]. Considering the linear association between maternal FPG levels and adverse pregnancy complications, there is no natural inflection point where the risk of adverse outcomes abruptly soars [12]. Sole reliance on arbitrarily defined diagnostic thresholds may occasionally be problematic, as medical interventions and patient awareness have improved [20]. Thus, associations between sub-diagnostic FPG levels and adverse perinatal outcomes are worth exploring.

Considering such circumstances, we conducted a retrospective cohort study to investigate the associations between FPG levels lower than the IADPSG diagnosis threshold during an OGTT and adverse maternal and infant outcomes in both GDM and non-GDM populations.

## Methods

This study is reported as per the Strengthening the Reporting of Observational Studies in Epidemiology (STROBE) guideline (S1 Checklist).

### Study design and participants

This single-center retrospective cohort study included data from patients seen from January 1, 2017, to May 31, 2022, at the Obstetrics and Gynecology Hospital of Fudan University, Shanghai, China. Eligible participants included women who received comprehensive obstetric care and underwent a 75-g OGTT between 24 and 28 weeks of gestation. During the study period, 36,059 singleton women were screened. Women were excluded for having incomplete OGTT results (*n* = 690) and pregnancy outcomes data (*n* = 509), severe hypertensive diseases (*n* = 301), late-onset GDM (*n* = 884), pregestational diabetes mellitus (*n* = 162), and overt diabetes mellitus complicating pregnancy (*n* = 28) [1]. In our study center, late-onset GDM was diagnosed when women had normal OGTT results at 24–28 gestational weeks but elevated FPG levels (≥5.1 mmol/L) at 32–34 gestational weeks during a routine antenatal blood testing [21]. The final cohort consisted of 33,417 women.

### Ethical approval

This study was approved by the ethics committee of Obstetrics and Gynecology Hospital of Fudan University on January 31st, 2024 (2021-90-X2). Informed consent was waived by the ethics committee due to the use of de-identified data in this retrospective study.

### Diagnosis of GDM and participant categorization

All participants underwent a 75-g OGTT between 24 and 28 weeks of gestation, with assessments of FPG, 1-h plasma glucose, and 2-h plasma glucose levels. A diagnosis of GDM was made if any of the following criteria were met: an FPG ≥5.1 mmol/L, a 1-h plasma glucose level ≥10.0 mmol/L, or a 2-h plasma glucose level ≥8.5 mmol/L [8]. Women diagnosed with GDM were further stratified into seven subgroups according to specific patterns of abnormal OGTT results. The subgroups were as follows: 0-h group: isolated abnormal FPG levels; 1-h group: an isolated abnormal 1-h plasma glucose level; 2-h group: an isolated abnormal 2-h plasma glucose level; 0-h + 1-h group: abnormal FPG and 1-h plasma glucose levels with a normal 2-h plasma glucose level; 0-h + 2-h group: abnormal FPG and 2-h plasma glucose levels with a normal 1-h plasma glucose level; 1-h + 2-h group: abnormal 1- and 2-h plasma glucose levels with normal FPG level; 0-h + 1-h + 2-h group: abnormal FPG, 1-h plasma glucose, and 2-h plasma glucose levels.

### Outcomes

The primary outcome was a composite of any of the following maternal and infant outcomes: gestational hypertension, preeclampsia, fetal death and stillbirth, preterm birth before 37 weeks of gestation, primary cesarean section, LGA, and small for gestational age (SGA). Gestational hypertension was defined as new-onset hypertension (either systolic blood pressure ≥140 mmHg or diastolic blood pressure ≥90 mmHg) after 20 weeks of gestation without systematic dysfunction. Preeclampsia was defined as new-onset hypertension accompanied by proteinuria or damage to other organ systems after 20 weeks of gestation [22]. Primary cesarean section was defined as a cesarean section performed for the first time. LGA and SGA were defined as birth weights greater than the 90th percentile or less than the 10th percentile for gestational age, respectively, on the basis of the Chinese population standard [23]. Compared with LGA and primary cesarean section, the incidences of gestational hypertension, preeclampsia, fetal death and stillbirth, preterm birth, and SGA were relatively low. However, these low-incidence complications often lead to more severe consequences. Therefore, a composite of these outcomes was introduced as hard endpoint in a post-hoc analysis.

### Statistical analysis

For baseline analysis, the mean and standard deviation were calculated for continuous variables, whereas absolute numbers and percentages were calculated for categorical variables. The absolute risk for any adverse outcome was estimated across the normal range of FPG and the full range of post-load plasma glucose during an OGTT. Absolute risks were calculated as the percentage of women with any adverse outcome and each individual outcome within each combination of FPG and post-load plasma glucose categories. Unweighted moving averages were calculated using a three-point window to smooth absolute risk estimates across FPG levels. For the boundary levels (≤3.8 mmol/L and ≥5.1 mmol/L), a two-point window was used. Generalized additive models with a binomial family were employed to explore the nonlinear relationship between lower FPG levels and any adverse outcome as well as each individual outcome, treating FPG levels as the independent variable and the outcome of interest as the dependent variable. Odds ratios (ORs) and 95% confidence intervals (CIs) for any adverse outcome and each individual outcome were calculated for GDM women with the same FPG level as non-GDM women. Primary analyses have been adjusted for maternal age, pre-pregnancy body mass index (BMI), ethnic group, educational level, and parity [3]. We have also applied splines of maternal age and pre-pregnancy BMI for adjustment considering their potential non-linear relationship with adverse perinatal outcomes. Statistical analysis was not planned before the study. All the statistical analyses were two-sided, with statistical significance considered at *P* < 0.05, and were performed via R (version 4.2.2). R packages (mgcv, ggplot2, and forestplot) were applied in the primary analysis and plotting [24–26].

## Results

### Participants

**Fig 1:**
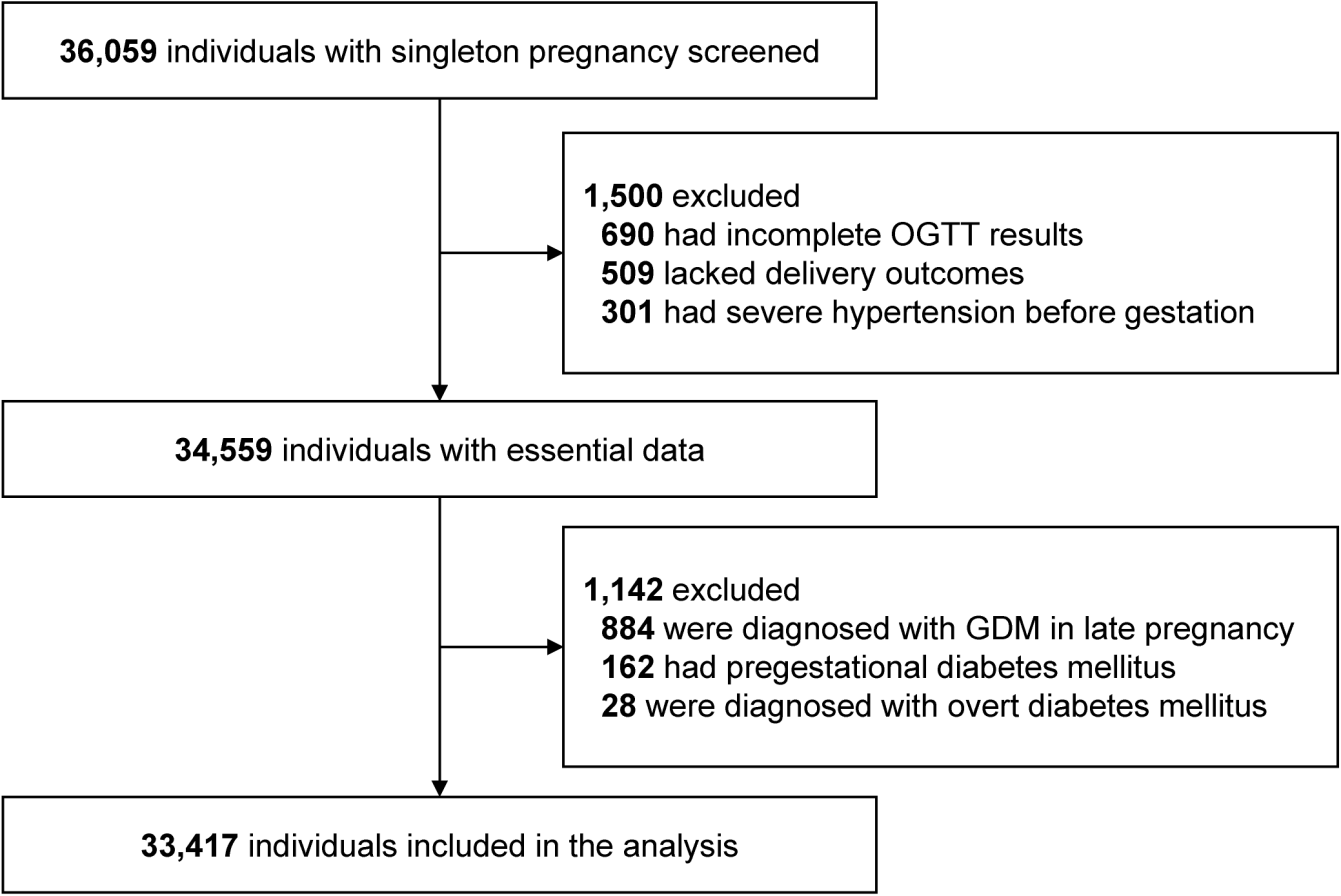
Flowchart of participant selection.

A total of 2,642 women were excluded based on the exclusion criteria leaving 33,417 women for analysis (Fig 1). Table 1 presented the sociodemographic characteristics of the study population and the incidence of perinatal outcomes among the different subgroups. The overall mean maternal age was 31.0 years, and the mean BMI was 21.2 kg/m^2^. The mean FPG, 1-h plasma glucose, and 2-h plasma glucose levels during the OGTT were 4.2, 7.3, and 6.2 mmol/L, respectively, and their quantiles were also provided in S1 Table. In general, the incidence of any adverse outcome was 49.9%, which was mainly dominated by primary cesarean delivery (27.3%) and LGA (20.6%). The incidences of other outcomes were 2.5% for gestational hypertension, 5.8% for preeclampsia, 0.1% for fetal death and stillbirth, 4.2% for preterm birth, and 3.6% for SGA. In terms of hard endpoint, the prevalence reached 14.5% in our study population. According to the IADPSG criteria, 3,180 (9.5%) participants were diagnosed with GDM, which were characterized by an advanced maternal age (32.2 versus 30.8 years, absolute difference: 1.4, 95% CI [1.25, 1.55], *p* < 0.001), a lower educational level, a higher BMI (22.3 versus 21.1 kg/m^2^, absolute difference: 1.16, 95% CI [1.04, 1.27], *p* < 0.001), a higher prevalence of multiparity (28.7% versus 24.7%, absolute difference: 4.0, 95% CI [2.3, 5.7], *p* < 0.001) and previous cesarean delivery (11.5% versus 8.6%, absolute difference: 3.0, 95% CI [1.8, 4.1], *p* < 0.001). About 17.6% (559/3,180) GDM women received insulin therapy after diagnosis. Compared with non-GDM women, the incidences of any adverse outcome (53.5% versus 48.9%, risk ratio [RR]: 1.09, 95% CI [1.06, 1.13], *p* < 0.001) and of hard endpoint (18.4% versus 14.1%, RR: 1.31, 95% CI [1.21, 1.42], *p* < 0.001) in GDM women were both significantly higher (Tables 1 and S2).

**Table 1:** Participants’ Socio-demographic Characteristics and Adverse Perinatal Outcomes (N = 33,417).

	Total (*N* = 33,417)	GDM (*n* = 3,180)^*^								Non-GDM (*n* = 30,237)
		All	0 h	1 h	2 h	0 h + 1 h	0 h + 2 h	1 h + 2 h	0 h + 1 h + 2 h	
**Subgroups, No. (%)** ^†^			404 (12.7)	909 (28.6)	895 (28.1)	140 (4.40)	46 (1.45)	622 (19.6)	164 (5.16)	
**Maternal age, mean (SD), y**	31.0 (4.0)	32.2 (4.1)	31.4 (3.9)	32.2 (4.1)	32.4 (4.2)	32.2 (4.5)	31.2 (4.1)	32.7 (4.0)	32.6 (4.1)	30.8 (3.9)
**Ethnic group, No. (%)**										
Han	32,987 (98.7)	3,145 (98.9)	402 (99.5)	894 (98.3)	887 (99.1)	140 (100.0)	46 (100.0)	613 (98.6)	163 (99.4)	29,842 (98.7)
Minority	430 (1.3)	35 (1.10)	2 (0.5)	15 (1.7)	8 (0.9)	0 (0.0)	0 (0.0)	9 (1.4)	1 (0.6)	395 (1.3)
**Education level, No. (%)**										
High school graduate or below	2,392 (7.2)	246 (7.7)	39 (9.7)	74 (8.1)	62 (6.9)	15 (10.7)	2 (4.3)	33 (5.3)	21 (12.8)	2,146 (7.1)
College graduate or above	20,190 (60.4)	1,804 (56.7)	205 (50.7)	548 (60.3)	511 (57.1)	64 (45.7)	26 (56.5)	369 (59.3)	81 (49.4)	18,386 (60.8)
Unknown/Other	10,835 (32.4)	1,130 (35.5)	160 (29.6)	287 (31.6)	322 (36.0)	61 (43.6)	18 (39.1)	220 (35.4)	62 (6.9)	9,705 (32.1)
**Pre-pregnancy weight, mean (SD), kg**	55.9 (8.2)	58.3 (9.4)	60.8 (10.3)	58.6 (9.5)	56.2 (8.3)	64.0 (12.0)	59.3 (10.7)	57.2 (8.2)	61.5 (9.5)	55.6 (8.0)
**Height, mean (SD), cm**	162 (5.0)	162 (5.1)	162.7 (5.0)	161.8 (5.2)	161.2 (5.0)	162.7 (4.7)	161.1 (4.8)	161.7 (4.9)	161.0 (5.5)	162.3 (5.0)
**BMI, mean (SD), kg/cm** ^**2**^ ^‡^	21.2 (2.8)	22.3 (3.23)	22.9 (3.5)	22.4 (3.2)	21.6 (2.9)	24.1 (4.0)	22.8 (4.0)	21.8 (2.8)	23.6 (3.1)	21.1 (2.8)
**BMI categories, No. (%)** ^§^										
<18.5	4,703 (14.1)	290 (9.1)	28 (6.9)	75 (8.3)	102 (11.4)	7 (5.0)	7 (15.2)	62 (10.0)	9 (5.5)	4,413 (14.6)
18.5–23.9	23,767 (71.1)	2,089 (65.7)	255 (63.1)	591 (65.0)	629 (70.3)	70 (50.0)	24 (52.1)	436 (70.1)	84 (51.2)	21,678 (71.7)
24–27.9	4,077 (12.2)	623 (19.6)	80 (19.8)	192 (21.1)	136 (15.2)	40 (28.6)	12 (26.1)	106 (17.0)	57 (34.8)	3,454 (11.4)
≥28	870 (2.6)	178 (5.6)	41 (10.1)	51 (5.6)	28 (3.1)	23 (16.4)	3 (6.5)	18 (2.9)	14 (8.5)	692 (2.3)
**Parity, No. (%)**										
Multigravida	8,368 (25.0)	912 (28.7)	107 (26.5)	254 (27.9)	266 (29.7)	46 (32.9)	13 (28.3)	178 (28.6)	48 (29.3)	7,456 (24.7)
Primigravida	25,037 (74.9)	2,268 (71.3)	297 (73.5)	655 (72.1)	629 (70.3)	94 (67.1)	33 (71.7)	444 (71.4)	116 (70.7)	22,769 (75.3)
Unknown	12 (0.04)	0 (0.00)	0 (0.0)	0 (0.0)	0 (0.0)	0 (0.0)	0 (0.0)	0 (0.0)	0 (0.0)	12 (0.04)
**Previous cesarean delivery, No. (%)**	2,957 (8.9)	367 (11.5)	47 (11.6)	108 (11.9)	107 (12.0)	22 (15.7)	6 (13.0)	55 (8.8)	22 (13.4)	2,590 (8.6)
**OGTT test in the second trimester, mean (SD), mmol/L**										
Fasting plasma glucose	4.2 (0.4)	4.6 (0.5)	5.2 (0.2)	4.4 (0.3)	4.3 (0.3)	5.4 (0.2)	5.2 (0.2)	4.5 (0.3)	5.5 (0.4)	4.2 (0.3)
1-h plasma glucose	7.3 (1.6)	9.9 (1.3)	8.3 (1.2)	10.5 (0.5)	8.9 (0.9)	11.0 (0.9)	9.1 (0.8)	10.8 (0.7)	11.4 (0.9)	7.1 (1.4)
2-h plasma glucose	6.2 (1.2)	8.2 (1.3)	6.7 (0.9)	7.3 (0.9)	9.0 (0.5)	7.3 (0.9)	9.2 (0.6)	9.3 (0.6)	9.6 (0.7)	6.0 (1.0)
**Insulin therapy, No. (%)**	–	559 (17.6)	73 (18.1)	113 (12.4)	111 (12.4)	46 (32.9)	13 (28.3)	133 (21.4)	70 (42.7)	–
**Types of adverse outcomes, No. (%)**										
Gestational hypertension	830 (2.5)	99 (3.11)	17 (4.2)	38 (4.2)	17 (1.9)	1 (0.7)	1 (2.2)	21 (3.4)	4 (2.4)	731 (2.4)
Preeclampsia	1,946 (5.8)	231 (7.26)	39 (9.7)	59 (6.5)	42 (4.7)	17 (12.1)	6 (13.0)	47 (7.6)	21 (12.8)	1,715 (5.7)
Fetal death and stillbirth	38 (0.1)	5 (0.16)	0 (0)	3 (0.33)	0 (0)	0 (0)	1 (2.17)	0 (0)	1 (0.6)	33 (0.1)
Preterm birth	1,392 (4.2)	186 (5.85)	31 (7.7)	42 (4.6)	49 (5.5)	11 (7.9)	6 (13.0)	31 (5.0)	16 (9.8)	1,206 (4.0)
Primary cesarean delivery	9,135 (27.3)	989 (31.1)	128 (31.7)	272 (30.0)	272 (30.4)	45 (32.1)	17 (37.0)	193 (31.0)	62 (37.8)	8,146 (26.9)
Small for gestational age	1,213 (3.6)	131 (4.12)	13 (3.2)	32 (3.5)	51 (5.7)	1 (0.7)	1 (2.2)	27 (4.3)	6 (3.7)	1,082 (3.6)
Large for gestational age	6,875 (20.6)	651 (20.5)	95 (23.5)	188 (20.7)	166 (18.5)	39 (27.9)	16 (34.8)	105 (16.9)	42 (25.6)	6,224 (20.6)
**Any adverse outcome, No. (%)** ^||^	16,493 (49.4)	1,702 (53.5)	230 (56.9)	477 (52.5)	453 (50.6)	86 (61.4)	30 (65.2)	320 (51.4)	106 (64.6)	14,791 (48.9)
**Hard endpoint, No. (%)** ^||^	4,836 (14.5)	586 (18.4)	87 (21.5)	155 (17.1)	144 (16.1)	27 (19.3)	12 (26.1)	115 (18.5)	46 (28.4)	4,250 (14.1)

* The GDM population was further categorized into seven subgroups based on the different combinations of elevated values during the OGTT: 0-h group: an isolated abnormal FPG level; 1-h group: an isolated abnormal 1-h plasma glucose (PG) level; 2-h group: an isolated abnormal 2-h PG level; 0-h + 1-h group: abnormal FPG and 1-h PG levels with a normal 2-h PG level; 0-h + 2-h group: abnormal FPG and 2-h PG levels with normal 1-h PG levels; 1-h + 2-h group: abnormal 1- and 2-h PG levels with a normal FPG level; 0-h + 1-h + 2-h group: abnormal FPG, 1-h PG, and 2-h PG levels.

† Calculated as the absolute number of women in each subgroup divided by the absolute number of women with GDM.

‡ Calculated as weight in kilograms divided by height in meters squared.

§ BMI cutoffs for the Chinese population.

|| Any adverse outcome was defined as the occurrence of any of the following outcomes: gestational hypertension, preeclampsia, fetal death or stillbirth, preterm birth, primary cesarean delivery, and small or large for gestational age. Hard endpoint was defined as the occurrence of any of the following outcomes: gestational hypertension, fetal death or stillbirth, preterm birth and small for gestational age.

BMI, body mass index; GDM, gestational diabetes mellitus; OGTT, oral glucose tolerance test; SD, standard deviation.

### OGTT results

The GDM population (*n* = 3,180) was further categorized into seven subgroups based on different combinations of elevated values during an OGTT: 0-h group: 404 women (12.7%); 1-h group: 909 women (28.6%); 2-h group: 895 women (28.1%);0-h + 1-h group: 140 women (4.4%); 0-h + 2-h group: 46 women (1.5%); 1-h + 2-h group: 622 women (19.6%); and 0-h + 1-h + 2-h group: 164 women (5.2%).

Overall, 76.3% (2,426 of 3,180) of the women with GDM had a normal FPG < 5.1 mmol/L (1-h, 2-h, and 1-h + 2-h groups). A lower incidence of adverse outcomes was observed among women with an isolated elevated OGTT value (0-h group: 56.9%, 1-h group: 52.5%, and 2-h group: 50.6%). Women with combined elevated OGTT values had a greater incidence of adverse outcomes (0-h + 1-h group: 61.4%, 0-h + 2-h group: 65.2%, and 0-h + 1-h + 2-h group: 64.6%), except for the 1-h + 2-h group (51.4%). Among the isolated elevation subgroups, women with elevated FPG levels (0-h group) presented the highest incidence of adverse outcomes (56.9%), followed by the 1-h (52.5%) and 2-h (50.6%) groups. Women with abnormal FPG levels ≥5.1 mmol/L had higher adverse outcome rates (0-h group: 56.9%, 0-h + 1-h group: 61.4%, 0-h + 2-h group: 65.2%, and 0-h + 1-h + 2-h group: 64.6%) than those with normal FPG levels (1-h group: 52.5%, 2-h group: 50.6%, and 1-h + 2-h group: 51.4%). Women with abnormal FPG levels had a higher incidence of preeclampsia, preterm birth, primary cesarean delivery, and LGA. Instead, women with normal FPG levels had a higher incidence of SGA, along with advanced maternal age, lower BMI, and fewer previous cesarean sections.

The distribution of FPG values within the normal range revealed distinct patterns between the GDM and non-GDM populations (S1 Fig). In the non-GDM population, FPG levels were skewed toward lower percentiles, ranging from 5% at the 90th percentile (P90, 4.6 mmol/L) to 15.8% at the P10 (3.8 mmol/L). Conversely, among GDM women with normal FPG levels, the distribution shifted toward higher percentiles, ranging from 4.6% at P20 (3.9 mmol/L) to 26.1% at P95 (4.8 mmol/L).

### Primary outcome

**Fig 2:**
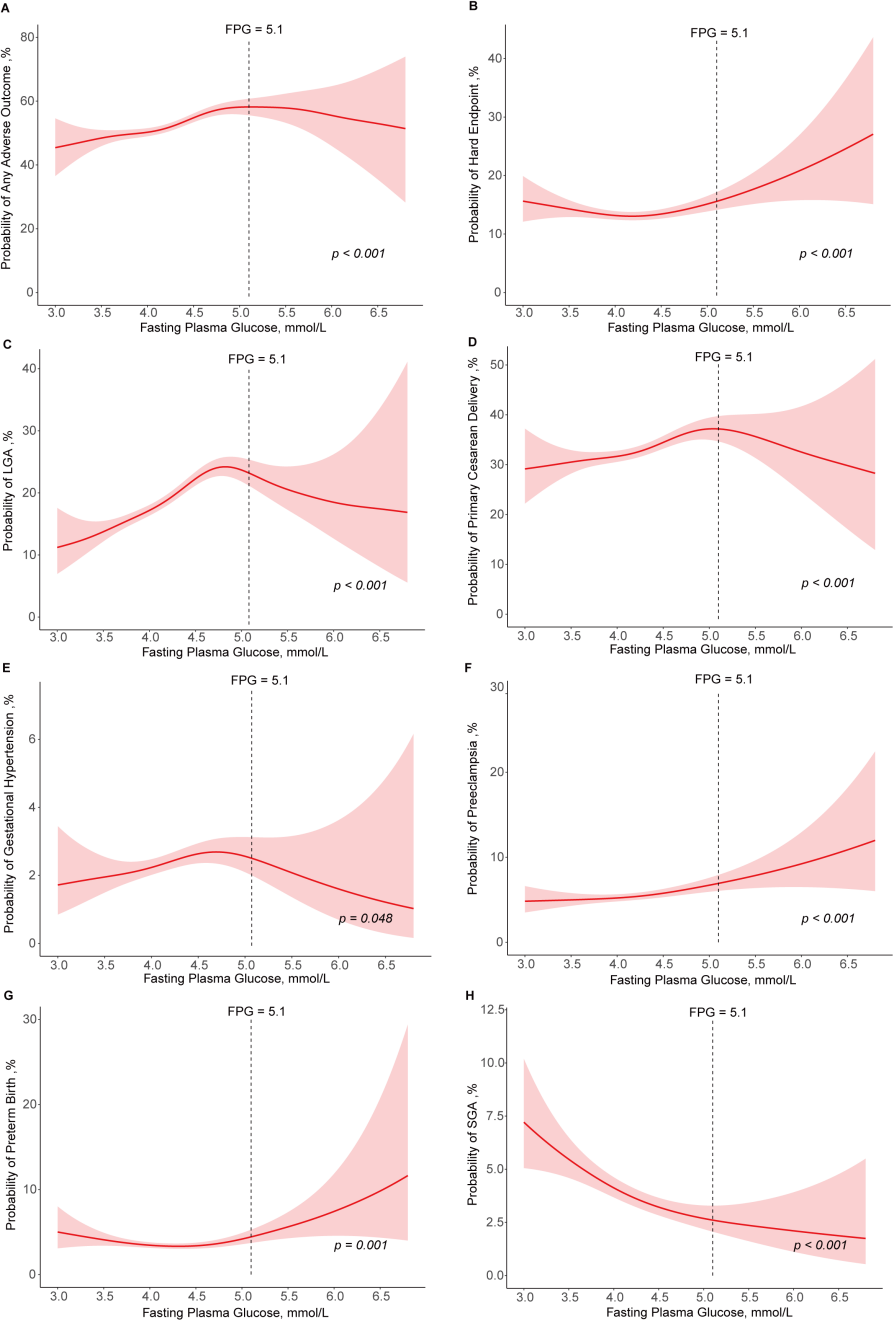
Modeled (observed) relationship between fpg levels and probability of adverse perinatal outcomes. **(A)** any adverse outcome, **(B)** hard endpoint, **(C)** LGA, **(D)** primary cesarean delivery, **(E)** gestational hypertension, **(F)** preeclampsia, **(G)** preterm birth, and **(H**) SGA. The vertical line at 5.1 mmol/L represented the GDM diagnostic threshold for FPG. FPG, fasting plasma glucose; LGA, large for gestational age; SGA, small for gestational age.

Across the full range of FPG levels, the probability of any adverse outcome in the total population increased linearly within the normal FPG levels but began to plateau and even slightly decline when FPG exceeded 5.1 mmol/L (Fig 2A). A similar trend was observed for LGA, gestational hypertension, and primary cesarean delivery. The association between FPG and the hard endpoint showed a U-shaped pattern, which was also seen in preterm birth. The risk of preeclampsia increased while risk of SGA decreased as FPG levels went higher (Fig 2B–2H).

For possible treatment effect, S3 Table showed the impact of insulin therapy on adverse outcomes. Among women who did not receive insulin treatment, FPG levels were linearly associated with increased risks of any adverse outcome, LGA, and primary cesarean delivery. In contrast, insulin-treated women exhibited comparable or slightly lower odds for these outcomes across FPG categories, suggesting a potential protective effect of insulin therapy for these common complications. However, this protective effect was not observed for the hard endpoint, where insulin therapy did not appear to significantly lower risk in women with elevated FPG levels.

**Fig 3:**
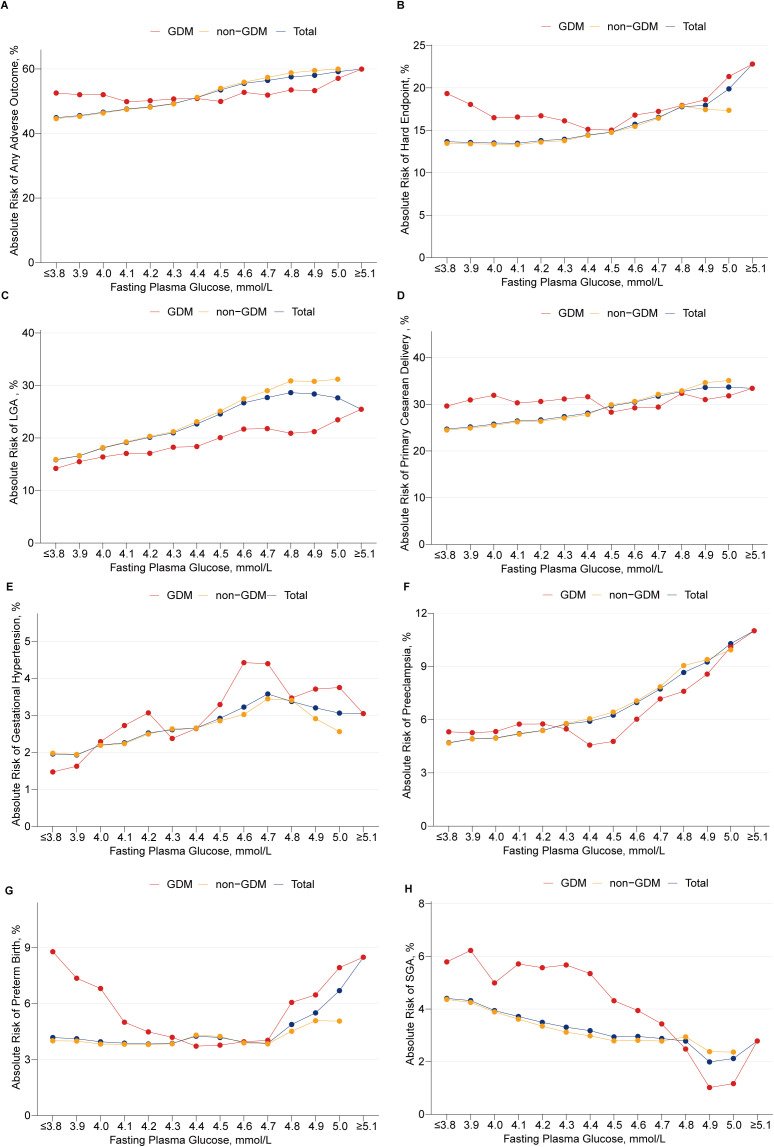
Absolute Risk for Adverse Perinatal Outcomes at different FPG Levels. The absolute risk at different FPG levels was calculated as the number of women with adverse outcomes divided by the number of women with every FPG level less than 5.1 mmol/L, multiplied by 100 in GDM, non-GDM, and total populations. The moving average was calculated at the window of three. The FPG interval was 0.1 mmol/L. **(A)** any adverse outcome, **(B)** hard endpoint, **(C)** LGA, **(D)** primary cesarean delivery, **(E)** gestational hypertension, **(F)** preeclampsia, **(G)** preterm birth, and **(H)** SGA. FPG, fasting plasma glucose; GDM, gestational diabetes mellitus; LGA, large for gestational age; SGA, small for gestational age.

Fig 3 showed the absolute risk of adverse outcomes at different FPG levels lower than 5.1 mmol/L in GDM, non-GDM, and total populations. The frequencies of adverse outcomes were presented in S2 Fig. As the FPG level changed, the risk curves were distinct between GDM and non-GDM populations. At lower FPG levels (<4.4 mmol/L), GDM women presented higher risk of any adverse outcome; however, as the FPG level exceeded 4.4 mmol/L and approached the diagnostic threshold, the risk was higher in non-GDM women. In the non-GDM population, the absolute risk of any adverse outcome steadily increased, reaching nearly 60% at the upper limit of FPG. Compared with non-GDM women, GDM women had a lower risk of LGA but a higher risk of SGA (Fig 3C and 3H).

**Fig 4:**
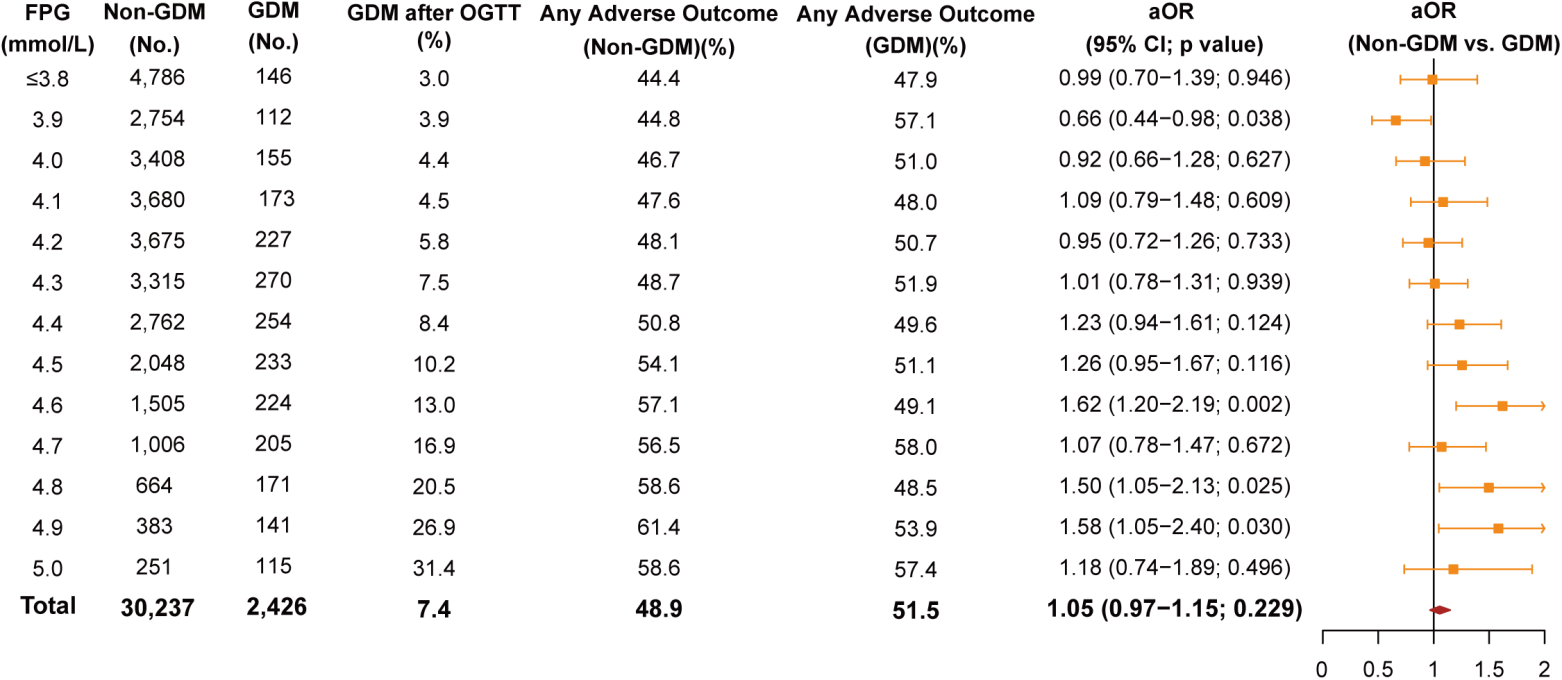
Adjusted odds ratios of any adverse outcome between women with and without GDM at different FPG levels. Adjusted OR indicated the odds ratio and it reflected the risk for any adverse outcome in non-GDM vs. GDM women at each FPG level, adjusting for maternal age, pre-pregnancy BMI, ethnic group, educational level, and parity. The solid circles represented the ORs for all participants at each FPG level. The error bars indicated 95% CIs. GDM after an OGTT indicated the diagnostic rate of GDM via OGTT results, which was calculated as the number of GDM women at each FPG level divided by the number of all the women at each FPG level. Any adverse outcome in the GDM population was calculated as the number of GDM women with any adverse outcome at each FPG level divided by the number of GDM women at each FPG level. Any adverse outcome in the non-GDM population was calculated as the number of non-GDM women with any adverse outcome at each FPG level divided by the number of non-GDM women at each FPG level. BMI, body mass index; CI, confidence interval; FPG, fasting plasma glucose; GDM, gestational diabetes mellitus; OGTT, oral glucose tolerance test.

Overall, any adverse outcome occurred in 51.5% (1,250 of 2,426) of the GDM population with normal FPG levels and in 48.9% (14,791/30,237) of the non-GDM population (Fig 4). As FPG level increased, an upward trend of GDM diagnosis was observed, ranging from 3.0% when lower than 3.8 mmol/L to 31.4% at 5.0 mmol/L. At higher FPG levels, non-GDM women faced significantly higher risks of any adverse outcome, with aORs of 1.62 (95% CI [1.20, 2.19]; *p* = 0.002) at 4.6 mmol/L, 1.50 (95% CI [1.05, 2.13]; *p* = 0.025) at 4.8 mmol/L, and 1.58 (95% CI [1.05, 2.40]; *p* = 0.030) at 4.9 mmol/L, respectively. Conversely, women without GDM had a significantly lower risk of any adverse outcome (aOR 0.66, 95% CI [0.44, 0.98]; *p* = 0.038 at 3.9 mmol/L) and of hard endpoint (aOR 0.63, 95% CI [0.42, 0.94; *p* = 0. 024 at ≤3.8 mmol/L) compared with their GDM counterparts with low fasting glycemia (Figs 4 and S3).

## Discussion

This retrospective cohort study investigated the associations between FPG levels lower than the IADPSG diagnostic threshold during an OGTT and a composite endpoint of poor perinatal outcome. Our findings revealed a continuous increase in the risk of adverse outcomes across the spectrum of FPG levels less than 5.1 mmol/L. Among women without IADPSG-defined GDM, those with borderline-normal FPG levels presented a greater risk of adverse outcomes. Conversely, women with IADPSG-defined GDM who experienced significant fluctuations in glucose levels were found to be at an elevated risk of adverse outcomes.

The HAPO study and subsequent studies identified a linear graded association between maternal blood glucose levels and complications affecting both mothers and offspring [7,17,27,28]. No natural inflection point at which risks of adverse outcomes significantly increase has been established [12]. The current exclusive reliance on dichotomized diagnostic thresholds not only directs disproportionate clinical attention toward diagnosed individuals but also may foster the misconception that lower blood glucose level correlate with better outcomes [29–31]. Few studies have investigated the relationship between lower blood glucose levels and diabetic complications.

Similar to previous research, our study findings have also raised concerns about the misleading ‘all-clear signal’ of negative diagnostic results [32,33]. Tennant and colleagues reported that women with a borderline FPG level (5.1–5.6 mmol/L) below the UK National Institute for Health and Care Excellent (NICE) criteria faced the highest risk of having LGA infants compared to women with NICE-defined GDM [15]. Our study showed that once the FPG level exceeded the IADPSG threshold and a diagnosis of GDM was made, the risk curve began to be flattening and slightly decreased with a further increase in the FPG level. This finding has provided two implications. First, women diagnosed with GDM tended to search for health services more actively and benefit from current GDM management strategies, including dietary control, physical activity, self-monitoring of blood glucose, and pharmacological interventions [30,34,35]. Second, among populations having the same level of FPG within normal range but near diagnostic threshold, non-GDM women were observed to experience greater risks of adverse outcomes than GDM women. Potential contributing factors may include suboptimal focus on glucose monitoring, dietary guidance, or weight management support during pregnancy, highlighting areas for improved clinical attention. However, the observed pattern in our risk curve could be partly due to regression to the mean, especially among women with FPG levels close to the diagnostic threshold. This possibility further reinforces the need for caution in interpreting outcome trends near diagnostic thresholds and highlights the importance of longitudinal glucose monitoring and individualized care strategies for all women with borderline glycemia. This population is more likely to develop hyperglycemia in late pregnancy, experience severe adverse complications, and even progress to type 2 diabetes mellitus later in life [18,32,33].

Our study suggested that women with lower FPG levels were not exempt from potential risks of adverse perinatal outcomes. We found that even within the normal range according to the IADPSG criteria, the risk of adverse outcomes remained linearly associated with increased FPG levels among all pregnant women. This association was particularly pronounced in women without GDM, where higher FPG and post-load plasma glucose levels correlated with a greater risk of adverse outcomes. Conversely, among women with GDM, the incidence of adverse outcomes was no longer strongly related to FPG or post-load plasma glucose levels but significant fluctuations in blood glucose levels (FPG < 4.4 mmol/L). A lower FPG level was associated with a lower possibility to be diagnosed with GDM, but once diagnosed with elevated PPG levels, they would have greater risks of adverse outcomes than non-GDM women. This finding indicated that fluctuations in the glycemic environment might also play a critical role in determining the risk of adverse perinatal outcomes among women with GDM, as reported in previous studies [36,37].

Although the three diagnostic thresholds proposed by the IADPSG are equally effective, previous studies have identified FPG levels as a stronger predictor for both perinatal and long-term complications than 1-hour or 2-hour plasma glucose levels [13,16,18]. Similarly, we observed that women with abnormal FPG levels presented a greater incidence of adverse perinatal outcomes than those with elevated post-load plasma glucose levels alone. Among the GDM population, women with abnormal FPG levels were less than 25% and had greater risk than those with normal FPG levels. Moreover, a distinct distribution pattern for FPG level within a normal range was identified between GDM and non-GDM populations in our study. The GDM population was clustered around individuals with higher FPG levels near the diagnostic threshold, whereas the non-GDM population was clustered around individuals with lower FPG levels. This finding indicated that GDM women, even those with normal FPG, exhibited potential dysglycemia at a baseline metabolic status. The underlying potential of FPG level for GDM screening and diagnosis as a plausible alternative to routine glucose tolerance testing for all pregnant women is worth further exploration [38,39].

However, our study has several limitations. First, in this single-center retrospective study conducted in Shanghai, the study population was limited to East Asian ancestry, who were identified with a higher prevalence of GDM based on elevated post-load glucose levels [7,40]. Applicability of the study results to other ethnicities could be limited considering different underlying pathophysiological mechanisms. Also, our study findings may not be fully generalizable to other less developed regions in China due to different lifestyles. Second, apart from insulin therapy, we did not further analyze the possible effects of other clinical interventions, such as nutrition consultation or physical activity, which can impact perinatal outcomes [41]. Third, we excluded women with late-onset GDM, which might represent a distinct perinatal complication in the high-risk subgroups identified in our study [33]. Additionally, the onset timing of part of pregnancy complications (i.e., gestational hypertensive diseases) could not be confirmed accurately due to the retrospective study design. The different patterns of association between FPG and each specific outcome might reflect underlying heterogeneity in pathophysiological mechanisms, but this hypothesis could not be tested with the current data. Finally, we did not analyze the associations between isolated 1-hour or 2-hour plasma glucose levels during OGTT and adverse maternal and infant outcomes. Future studies are encouraged to include more representative populations and comprehensive infant outcome data to enhance the understanding of these associations.

In summary, this study highlighted that the association between maternal FPG levels and adverse perinatal outcomes was not only significant but also presented different patterns for women with and without GDM, even when the FPG level was lower than the widely endorsed IADPSG threshold. These findings caution against sole reliance on the one-size-fits-all diagnostic criteria and underscore the need for individualized monitoring and management for women with different glucose levels.

## Supporting information

S1 Fig Distribution of FPG levels lower than the IADPSG diagnostic threshold in GDM and non-GDM populations. (DOCX)

S2 Fig Frequency in adverse perinatal outcomes at different FPG levels. (DOCX)

S3 Fig Odds ratios of any adverse outcome and adjusted odds ratios of hard endpoint between women with and without GDM at different FPG levels. (DOCX)

S1 Table Quantiles of plasma glucose levels at OGTT. (DOCX)

S2 Table Effect sizes of baseline characteristics and adverse outcomes between GDM and non-GDM women. (DOCX)

S3 Table The effect of insulin therapy on risk of adverse outcomes associated with different levels of FPG at OGTT. (DOCX)

S1 Checklist STROBE checklist. (DOCX)

## Abbreviations

aOR: adjusted odds ratio
BMI: body mass index
CI: confidence interval
FPG: fasting plasma glucose
GDM: gestational diabetes mellitus
HAPO: hyperglycemia and adverse pregnancy outcomes
IADPSG: International Association of Diabetes and Pregnancy Study Groups
LGA: large for gestational age
NICE: National Institute for Health and Care Excellent
OGTT: oral glucose tolerance test
OR: odds ratio
RR: risk ratio
SGA: small for gestational age
