# Supplementary material for: Association between lower fasting plasma glucose levels during oral glucose tolerance test and adverse perinatal outcomes: A Chinese cohort study
Source: PLoS Med. 2025 Sep 23;22(9):e1004722. doi: 10.1371/journal.pmed.1004722 (PMC12456778; doi:10.1371/journal.pmed.1004722)
Supplement: S1 Fig — (DOCX) [file pmed.1004722.s001.docx]

**S1 Fig. Distribution of FPG Levels Lower than the IADPSG Diagnostic Threshold in GDM and non-GDM Populations.**

**
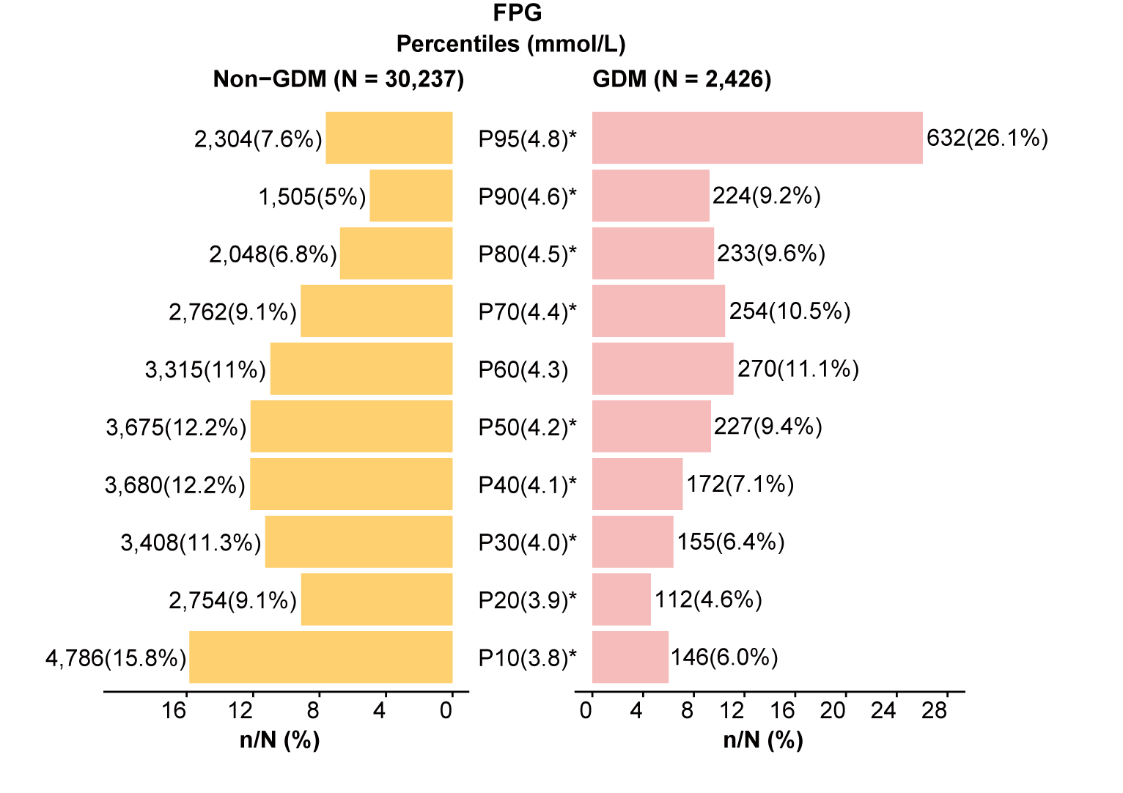
**

The FPG levels lower than the IADPSG diagnostic threshold (5.1 mmol/L) were divided by percentiles. The percentage of individuals at each FPG percentile level was calculated as the number of GDM or non-GDM individuals at each FPG percentile divided by the number of GDM or non-GDM individuals with FPG levels < 5.1 mmol/L. Chi-square test was applied for the statistical comparison between number of individuals at each FPG level (* p-value < 0.05).

FPG, fasting plasma glucose; GDM, gestational diabetes mellitus; IADPSG, International Association of Diabetes and Pregnancy Study Groups.
