# Supplementary material for: Association between lower fasting plasma glucose levels during oral glucose tolerance test and adverse perinatal outcomes: A Chinese cohort study
Source: PLoS Med. 2025 Sep 23;22(9):e1004722. doi: 10.1371/journal.pmed.1004722 (PMC12456778; doi:10.1371/journal.pmed.1004722)
Supplement: S2 Fig — (DOCX) [file pmed.1004722.s002.docx]

**S2 Fig. Frequency in Adverse Perinatal Outcomes at Different FPG Levels.**


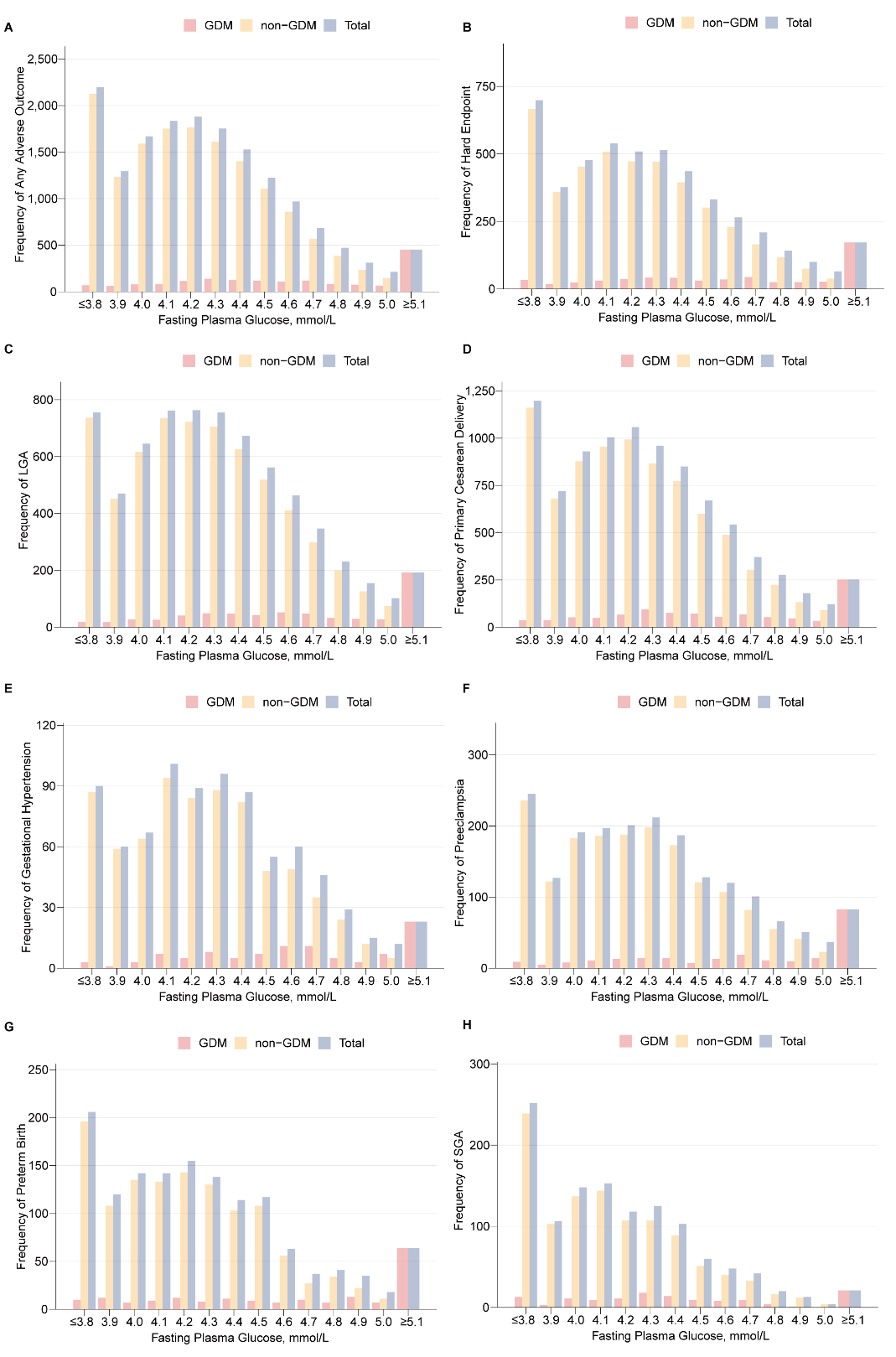


The frequency represented the number of women with adverse outcomes in GDM, non-GDM, and total populations at different FPG levels less than 5.1 mmol/L. The FPG interval was 0.1 mmol/L. (A) any adverse outcome, (B) hard endpoint, (C) LGA, (D) primary cesarean delivery, (E) gestational hypertension, (F) preeclampsia, (G) preterm birth, and (H) SGA.

FPG, fasting plasma glucose; GDM, gestational diabetes mellitus; LGA, large for gestational age; SGA, small for gestational age.
