# Supplementary material for: Association between lower fasting plasma glucose levels during oral glucose tolerance test and adverse perinatal outcomes: A Chinese cohort study
Source: PLoS Med. 2025 Sep 23;22(9):e1004722. doi: 10.1371/journal.pmed.1004722 (PMC12456778; doi:10.1371/journal.pmed.1004722)
Supplement: S3 Fig — (DOCX) [file pmed.1004722.s003.docx]

**S3 Fig. Odds Ratios of Any Adverse Outcome and Adjusted Odds Ratios of Hard Endpoint between Women with and without GDM at Different FPG Levels.**

**
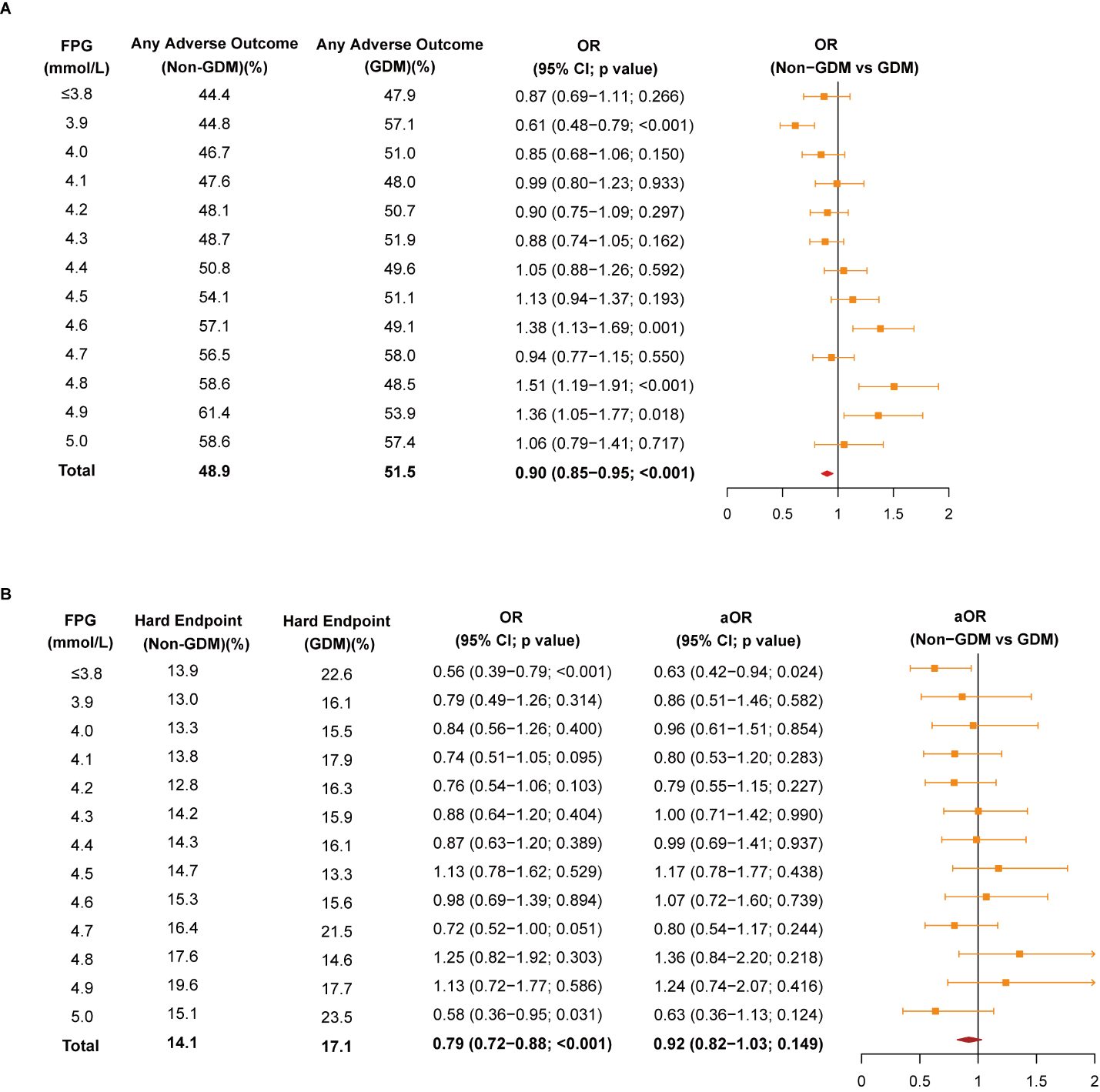
**

The OR indicated the odds ratio and it reflected the risk for any adverse outcome in non-GDM versus GDM women at each FPG level (A). The OR indicated the odds ratio and it reflected the risk for the hard endpoint in non-GDM versus GDM women at each FPG level. The adjusted ORs were adjusted for maternal age, pre-pregnancy BMI, ethnic group, educational level, and parity (B). The solid circles represent the ORs for all participants at each FPG level. The error bars indicate 95% confidence intervals. The outcome in the GDM population was calculated as the number of GDM women with the outcome at each FPG level divided by the number of GDM women at each FPG level. The outcome in the non-GDM population was calculated as the number of non-GDM women with the outcome at each FPG level divided by the number of non-GDM women at each FPG level.

aOR, adjusted odds ratio; BMI, body mass index; CI, confidence interval; FPG, fasting plasma glucose; GDM, gestational diabetes mellitus; OR, odds ratio.
