# Supplementary material for: Association between lower fasting plasma glucose levels during oral glucose tolerance test and adverse perinatal outcomes: A Chinese cohort study
Source: PLoS Med. 2025 Sep 23;22(9):e1004722. doi: 10.1371/journal.pmed.1004722 (PMC12456778; doi:10.1371/journal.pmed.1004722)
Supplement: S1 Table — (DOCX) [file pmed.1004722.s004.docx]

**S1 Table. Quantiles of Plasma Glucose Levels at OGTT**

|  | **All women** | **GDM women** | **Non-GDM women** |
| --- | --- | --- | --- |
| **FPG,**  **median (q1, q3), mmol/L** | 4.2 (4.0, 4.4) | 4.6 (4.2, 5.0) | 4.0 (4.2, 4.4) |
| **1-hour plasma glucose, median (q1, q3), mmol/L** | 7.3 (6.2, 8.4) | 10.1 (9.2, 10.7) | 7.1 (6.1, 8.1) |
| **2-hour plasma glucose, median (q1, q3), mmol/L** | 6.1 (5.4, 7.0) | 8.5 (7.4, 9.1) | 6.0 (5.3, 6.7) |

FPG, fasting plasma glucose; GDM, gestational diabetes mellitus; OGTT, oral glucose tolerance test.
