# Supplementary material for: Association between lower fasting plasma glucose levels during oral glucose tolerance test and adverse perinatal outcomes: A Chinese cohort study
Source: PLoS Med. 2025 Sep 23;22(9):e1004722. doi: 10.1371/journal.pmed.1004722 (PMC12456778; doi:10.1371/journal.pmed.1004722)
Supplement: S2 Table — (DOCX) [file pmed.1004722.s005.docx]

**S2 Table.** **Effect Sizes of Baseline Characteristics and Adverse Outcomes between GDM and non-GDM Women**

| **Types of adverse outcomes, No. (%)** | **GDM** | **Non-GDM** | **Absolute Difference**  **(95% CI)** | **P value** | **RR^*^ (95% CI)** | **P value** |
| --- | --- | --- | --- | --- | --- | --- |
| **Age, mean (SD), years** | 32.2 (4.1) | 30.8 (3.9) | 1.4 (1.25 - 1.55) | < 0.001 | **--** | **--** |
| **Pre-pregnancy BMI, mean (SD), kg/m^2^** | 22.3 (3.2) | 21.1 (2.8) | 1.16 (1.04 - 1.27) | < 0.001 | **--** | **--** |
| **Multigravida, No. (%)** | 912 (28.7) | 7,456 (24.7) | 4.0 (2.3 - 5.7) | < 0.001 | **--** | **--** |
| **Previous cesarean delivery****, No. (%)** | 367 (11.5) | 2,590 (8.6） | 3.0 (1.8 - 4.1) | < 0.001 | **--** | **--** |
| **Gestational hypertension, No. (%)** | 99 (3.11) | 731 (2.4) | 0.7 (0.1 - 1.3) | 0.019 | 1.29 (1.05 - 1.58) | 0.016 |
| **Preeclampsia, No. (%)** | 231 (7.26) | 1,715 (5.7) | 1.6 (0.7 - 2.5) | < 0.001 | 1.28 (1.12 - 1.46) | < 0.001 |
| **Fetal death and stillbirth, No. (%)** | 5 (0.16) | 33 (0.1) | 0.0 (-0.1 - 0.2) | 0.625 | 1.44 (0.56 - 3.69) | 0.45 |
| **Preterm birth, No. (%)** | 186 (5.85) | 1,206 (4.0) | 1.9 (1 - 2.7) | < 0.001 | 1.47 (1.26 - 1.70) | < 0.001 |
| **Primary cesarean delivery, No. (%)** | 989 (31.1) | 8,146 (26.9) | 4.2 (2.5 - 5.8) | < 0.001 | 1.15 (1.09 - 1.22) | < 0.001 |
| **Small for gestational age, No. (%)** | 131 (4.12) | 1,082 (3.6) | 0.5 (-0.2 - 1.3) | 0.13 | 1.15 (0.96 - 1.37) | 0.12 |
| **Large for gestational age, No. (%)** | 651 (20.5) | 6,224 (20.6) | -0.1 (-1.6 - 1.4) | 0.90 | 0.99 (0.93 - 1.07) | 0.88 |
| **Any adverse outcome, No. (%)** | 1,702 (53.5) | 14,791 (48.9) | 4.6 (2.8 - 6.4) | < 0.001 | 1.09 (1.06 - 1.13) | < 0.001 |
| **Hard endpoint, No. (%)** | 586 (18.4) | 4,250 (14.1) | 4.4 (3 - 5.8) | < 0.001 | 1.31 (1.21 - 1.42) | < 0.001 |

^*^ Poisson regression model was applied.

BMI, body mass index; CI, confidence interval; GDM, gestational diabetes mellitus; RR, risk ratio.
