# Supplementary material for: Association between lower fasting plasma glucose levels during oral glucose tolerance test and adverse perinatal outcomes: A Chinese cohort study
Source: PLoS Med. 2025 Sep 23;22(9):e1004722. doi: 10.1371/journal.pmed.1004722 (PMC12456778; doi:10.1371/journal.pmed.1004722)
Supplement: S3 Table — (DOCX) [file pmed.1004722.s006.docx]

**S3 Table. The Effect of Insulin Therapy on Risk of Adverse Outcomes Associated with Different Levels of FPG at OGTT.**

|  |  | **Present study** | | | | | **HAPO** |
| --- | --- | --- | --- | --- | --- | --- | --- |
| **FPG categories^*^** | **FPG values (mmol/L)** | **No. (%)** | **OR (95% CI)** | **P value** | **Adjusted OR^†^ (95% CI)** | **P value** | **Adjusted OR (95% CI)** |
| **Any adverse outcome** | | | | | | | |
| 1 | < 4.2 | 6,703 (45.8) | 1(reference) | / | 1(reference) | / | / |
| 2 | 4.2-4.4 | 4,782 (49.0) | 1.14(1.08-1.20) | < 0.001 | **1.08(1.02-1.13)** | < 0.001 | / |
| 3 | 4.5-4.7 | 2,535 (55.6) | 1.48(1.39-1.58) | < 0.001 | **1.35(1.26-1.44)** | < 0.001 | / |
| 4 | 4.8-4.9 | 624 (59.6) | 1.74(1.54-1.98) | < 0.001 | **1.49(1.31-1.70)** | < 0.001 | / |
| 5 | 5.0-5.2 | 222 (60.2) | 1.79(1.45-2.21) | < 0.001 | **1.42(1.14-1.76)** | < 0.001 | / |
| 6 | 5.3-5.5 | 42 (63.6) | 2.07(1.26-3.47) | < 0.001 | 1.42(0.85-2.42) | 0.184 | / |
| 7 | ≥ 5.6 | 35 (66.0) | 2.30(1.32-4.15) | < 0.001 | 1.46(0.83-2.69) | 0.202 | / |
| **Hard endpoint** | | | | | | | |
| 1 | < 4.2 | 1,986 (13.6) | 1(reference) | / | 1(reference) | / | 1(reference) |
| 2 | 4.2-4.4 | 1,338 (13.7) | 1.01(0.94-1.09) | 0.749 | 0.95(0.88-1.02) | 0.176 | / |
| 3 | 4.5-4.7 | 696 (15.3) | 1.15(1.04-1.26) | 0.004 | 1.02(0.92-1.12) | 0.734 | / |
| 4 | 4.8-4.9 | 192 (18.3) | 1.43(1.21-1.68) | < 0.001 | **1.19(1.00-1.40)** | 0.045 | / |
| 5 | 5.0-5.2 | 73 (19.8) | 1.57(1.20-2.02) | < 0.001 | 1.19(0.91-1.54) | 0.202 | / |
| 6 | 5.3-5.5 | 18 (27.3) | 2.39(1.35-4.04) | 0.002 | 1.55(0.87-2.67) | 0.123 | / |
| 7 | ≥ 5.6 | 17 (32.1) | 3.01(1.65-5.28) | < 0.001 | **1.84(1.00-3.28)** | 0.045 | / |
| **LGA** | | | | | | | |
| 1 | < 4.2 | 2,542 (17.4) | 1(reference) | / | 1(reference) | / | 1(reference) |
| 2 | 4.2-4.4 | 2,054 (21.1) | 1.27(1.19-1.35) | < 0.001 | **1.21(1.13-1.29)** | < 0.001 | 1.37(1.16-1.62) |
| 3 | 4.5-4.7 | 1,229 (27.0) | 1.75(1.62-1.90) | < 0.001 | **1.59(1.47-1.72)** | < 0.001 | 1.72(1.46-2.03) |
| 4 | 4.8-4.9 | 325 (31.0) | 2.14(1.86-2.45) | < 0.001 | **1.85(1.61-2.13)** | < 0.001 | 1.95(1.62-2.35) |
| 5 | 5.0-5.2 | 99 (26.8) | 1.74(1.37-2.20) | < 0.001 | **1.35(1.06-1.71)** | < 0.001 | 2.73(2.25-3.31) |
| 6 | 5.3-5.5 | 15 (22.7) | 1.40(0.758-2.43) | 0.255 | 0.90(0.483-1.58) | 0.532 | 3.00(2.34-3.86) |
| 7 | ≥ 5.6 | 15 (22.7) | 2.45(1.35-4.27) | < 0.001 | 1.48(0.807-2.63) | 0.636 | 5.01(3.54-7.09) |
| **Primary Cesarean Delivery** | | | | | | | |
| 1 | < 4.2 | 3,675 (25.1) | 1(reference) | / | 1(reference) | / | 1(reference) |
| 2 | 4.2-4.4 | 2,634 (27.0) | 1.10(1.04-1.17) | < 0.001 | 1.05(0.99-1.12) | 0.124 | 1.19 (1.06-1.34) |
| 3 | 4.5-4.7 | 1,391 (30.5) | 1.31(1.22-1.41) | < 0.001 | **1.23(1.14-1.33)** | < 0.001 | 1.21 (1.07-1.37) |
| 4 | 4.8-4.9 | 357 (34.1) | 1.54(1.35-1.76) | < 0.001 | **1.37(1.18-1.57)** | < 0.001 | 1.33 (1.15-1.54) |
| 5 | 5.0-5.2 | 137 (37.1) | 1.76(1.42-2.18) | < 0.001 | **1.53(1.21-1.92)** | < 0.001 | 1.44 (1.23-1.69) |
| 6 | 5.3-5.5 | 26 (39.4) | 1.94(1.17-3.16) | < 0.001 | 1.56(0.912-2.64) | 0.098 | 1.39 (1.11-1.75) |
| 7 | ≥ 5.6 | 17 (32.1) | 1.41(0.77-2.47) | 0.247 | 1.07(0.570-1.95) | 0.821 | 1.60 (1.12-2.27) |

^*^ The FPG category was referred to HAPO study. In the present study, only non-GDM women were included in category 1 to 4; only GDM women who received insulin therapy were included in category 6 to 7; part of non-GDM and insulin-treated GDM women were included in category 5.

**^†^** The ORs were adjusted for maternal age, pre-pregnancy BMI, ethnic group, educational level, and parity.

CI, confidence interval; FPG, fasting plasma glucose; HAPO, hyperglycemia and adverse pregnancy outcomes; LGA, large for gestational age; OGTT, oral glucose tolerance test; OR, odds ratio.
