# Supplementary material for: Association between lower fasting plasma glucose levels during oral glucose tolerance test and adverse perinatal outcomes: A Chinese cohort study
Source: PLoS Med. 2025 Sep 23;22(9):e1004722. doi: 10.1371/journal.pmed.1004722 (PMC12456778; doi:10.1371/journal.pmed.1004722)
Supplement: S1 Checklist — (DOCX) [file pmed.1004722.s007.docx]

**S1 Checklist. STROBE Checklist**

|  | Item No | Recommendation | Page No |
| --- | --- | --- | --- |
| **Title and abstract** | 1 | (*a*) Indicate the study’s design with a commonly used term in the title or the abstract |  |
|  |  | (*b*) Provide in the abstract an informative and balanced summary of what was done and what was found | Title in Page 1;  Abstract in Page 3 |
| Introduction | | | |
| Background/rationale | 2 | Explain the scientific background and rationale for the investigation being reported | Paragraphs 1-3 in Introduction (Page 6-7) |
| Objectives | 3 | State specific objectives, including any prespecified hypotheses | Paragraph 4 in Introduction (Page 7) |
| Methods | | | |
| Study design | 4 | Present key elements of study design early in the paper | Paragraph 1 in Study Design and Participants section (Page 7-8) |
| Setting | 5 | Describe the setting, locations, and relevant dates, including periods of recruitment, exposure, follow-up, and data collection | Paragraph 1 in Study Design and Participants section (Page 7-8) |
| Participants | 6 | (*a*) Give the eligibility criteria, and the sources and methods of selection of participants. Describe methods of follow-up | S1 Fig in Supporting Information |
|  |  | (*b*) For matched studies, give matching criteria and number of exposed and unexposed |  |
| Variables | 7 | Clearly define all outcomes, exposures, predictors, potential confounders, and effect modifiers. Give diagnostic criteria, if applicable | Paragraph 1 in Outcomes section (Page 9) |
| Data sources/ measurement | 8* | For each variable of interest, give sources of data and details of methods of assessment (measurement). Describe comparability of assessment methods if there is more than one group | Paragraphs 4-5 in Methods section (Page 8-9) |
| Bias | 9 | Describe any efforts to address potential sources of bias | Paragraph 1 in Statistical Analysis section (Page 10) |
| Study size | 10 | Explain how the study size was arrived at | S1 Fig in Supporting Information,  Paragraph 1 in Results section (Page 10) |
| Quantitative variables | 11 | Explain how quantitative variables were handled in the analyses. If applicable, describe which groupings were chosen and why | Paragraph 1 in Statistical Analysis section (Page 11) |
| Statistical methods | 12 | (*a*) Describe all statistical methods, including those used to control for confounding |  |
|  |  | (*b*) Describe any methods used to examine subgroups and interactions |  |
|  |  | (*c*) Explain how missing data were addressed | Paragraph 1 in Statistical Analysis section (Page 10) |
|  |  | (*d*) If applicable, explain how loss to follow-up was addressed |  |
|  |  | (*e*) Describe any sensitivity analyses |  |
| Results | | |  |
| Participants | 13* | (a) Report numbers of individuals at each stage of study—eg numbers potentially eligible, examined for eligibility, confirmed eligible, included in the study, completing follow-up, and analysed | Paragraph 1 in Results section (Page 11), S1 Fig in Supporting Information |
|  |  | (b) Give reasons for non-participation at each stage |  |
|  |  | (c) Consider use of a flow diagram |  |
| Descriptive data | 14* | (a) Give characteristics of study participants (eg demographic, clinical, social) and information on exposures and potential confounders |  |
|  |  | (b) Indicate number of participants with missing data for each variable of interest | Paragraph 1 in Results section (Page 11), Table 1 |
|  |  | (c) Summarise follow-up time (eg, average and total amount) |  |
| Outcome data | 15* | Report numbers of outcome events or summary measures over time | Paragraph 1 in Results section (Page 11), Table 1 |

| Main results | 16 | (*a*) Give unadjusted estimates and, if applicable, confounder-adjusted estimates and their precision (eg, 95% confidence interval). Make clear which confounders were adjusted for and why they were included | Paragraphs 1-4 in Primary Outcome section (Page 15-17), Figs 1-3 |
| --- | --- | --- | --- |
|  |  | (*b*) Report category boundaries when continuous variables were categorized |  |
|  |  | (*c*) If relevant, consider translating estimates of relative risk into absolute risk for a meaningful time period |  |
| Other analyses | 17 | Report other analyses done—eg analyses of subgroups and interactions, and sensitivity analyses | S2-S4 Figs, S1-S3 Tables |
| Discussion | | | |
| Key results | 18 | Summarise key results with reference to study objectives | Paragraph 1 in Discussion section (Page 18) |
| Limitations | 19 | Discuss limitations of the study, taking into account sources of potential bias or imprecision. Discuss both direction and magnitude of any potential bias | Paragraph 6 in Discussion section (Page 21) |
| Interpretation | 20 | Give a cautious overall interpretation of results considering objectives, limitations, multiplicity of analyses, results from similar studies, and other relevant evidence | Paragraphs 2-5 in Discussion section (Page 18-21) |
| Generalisability | 21 | Discuss the generalisability (external validity) of the study results | Paragraphs 6 and 7 in Discussion section (Page 21-22) |
| Other information | | | |
| Funding | 22 | Give the source of funding and the role of the funders for the present study and, if applicable, for the original study on which the present article is based | Financial Disclosure Statement in Acknowledgements (Page 22-23) |

*Give information separately for exposed and unexposed groups.
